# ENABLE—App-Based Digital Capture and Intervention of Patient-Reported Quality of Life, Adverse Events, and Treatment Satisfaction in Breast Cancer: Protocol for a Randomized Controlled Trial

**DOI:** 10.2196/69855

**Published:** 2025-05-26

**Authors:** Thomas M Deutsch, Léa L Volmer, Manuel Feisst, Laura Bodenbeck, Kathrin Hassdenteufel, Lara Tretschock, Christiane Breit, Stefan Stefanovic, Armin Bauer, Carolin Anders, Lina Weinert, Tobias Engler, Andreas D Hartkopf, Nico Pfeifer, Pascal Escher, Marc Mausch, Oliver Heinze, Marc Suetterlin, Sara Y Brucker, Andreas Schneeweiss, Markus Wallwiener

**Affiliations:** 1 Department of Gynecology and Obstetrics Heidelberg University Hospital Heidelberg Germany; 2 Department of Gynecology and Obstetrics University Hospital Tuebingen Tuebingen Germany; 3 Institute of Medical Biometry Heidelberg University Heidelberg Germany; 4 Department of Gynecology and Obstetrics University Hospital Mannheim, Heidelberg University Mannheim Germany; 5 Institute Women's Health Tuebingen Germany; 6 Institute of Medical Informatics Heidelberg University Heidelberg Germany; 7 Directorate of Nursing Heidelberg University Hospital Heidelberg Germany; 8 Heidelberg Institute of Global Health, Section for Oral Health Heidelberg University Heidelberg Germany; 9 Department of Computer Science University of Tuebingen Tuebingen Germany; 10 Center for Innovative Care University Hospital Tuebingen Tuebingen Germany; 11 Institute for Bioinformatics and Medical Informatics University of Tuebingen Tuebingen Germany; 12 Rhein Main University of Applied Sciences Wiesbaden Germany; 13 National Center for Tumor Diseases University Hospital Heidelberg Heidelberg Germany; 14 German Cancer Research Center Heidelberg Germany; 15 Department of Gynecology and Obstetrics University Hospital Halle Halle Germany

**Keywords:** breast cancer, electronic patient-reported outcomes, ePRO, health-related quality of life, HRQoL, eHealth apps

## Abstract

**Background:**

In recent years, breast cancer treatment has taken the path toward personalized medicine. Based on individual tumor biology, therapy tailored to the particular subtype of cancer is increasingly being used. The aim here is to find the most suitable therapy for the disease. However, the success of therapy depends to a large extent on the patient’s adherence to treatment. This, in turn, depends on how the therapy is tolerated and how the treatment team cares for the patient. Patient-centered care seeks to identify and address the individual needs of each patient and to find the best form of care for that person.

**Objective:**

In order to improve comprehensive oncological care of patients with breast cancer, the ENABLE trial digitally recorded the health-related quality of life (HRQoL), adverse events (AEs), and patient satisfaction using a mobile smartphone app. The trial provided individualized responses to reported AEs and offered assistance. Additionally, it assessed the impact of a patient-reported outcome–based intervention across various therapy settings.

**Methods:**

Patients with breast cancer were eligible to participate in the study before neoadjuvant, adjuvant, postneoadjuvant, or palliative systemic therapy against breast cancer was initiated at the Heidelberg, Mannheim, and Tuebingen, Germany, university hospitals. After 1:1 randomization into an intervention and a control group, HRQoL assessments were performed at six fixed time points during the therapy using validated questionnaires. In the intervention group, HRQoL was also assessed briefly every week using a visual analog scale (EQ-VAS). In cases of significant deterioration, therapy-associated side effects were assessed in a graduated manner, recommendations were sent to the patient, and the treatment team was informed. Additionally, the app served as an “eHealth companion” for education, training, and organizational support during therapy.

**Results:**

Recruitment started in March 2021; follow-up was completed in February 2024. In total, 606 patients were enrolled, and 592 patients participated in the study. Enrollment was completed in September 2023, and the last visit was in February 2024. The first results are expected to be published in Q2 2025.

**Conclusions:**

Participation in the intervention group is expected to improve treatment satisfaction, adherence, detection, and timely treatment of critical AEs. The close-meshed, weekly, brief HRQoL assessment will also be tested as a screening tool to detect relevant side effects during therapy. The study offers a more objective HRQoL assessment across treatment strategies.

**Trial Registration:**

Deutsches Register Klinischer Studien DRKS00025611; https://drks.de/search/en/trial/DRKS00025611

**International Registered Report Identifier (IRRID):**

DERR1-10.2196/69855

## Introduction

Breast cancer is the most common cancer in women worldwide and the fifth leading cause of cancer-related deaths [1]. In recent years, oncological therapy, in general, and treatment of breast cancer, in particular, have taken the path toward personalized medicine [2]. Current treatment of both primary and advanced breast cancer is individualized and tailored to each patient’s tumor subtype and needs. The aim here is to find the most suitable therapy. However, the success of therapy depends to a large extent on the patient’s adherence to treatment [3,4]. This, in turn, depends on how the therapy is tolerated and how the medical team cares for the patient [5]. One approach is to personalize patient care overall. Therefore, a patient’s individual needs must be identified and taken into account with the aim of finding the best form of care for each person. Patient-centered care “is respectful of and responsive to individual patient preferences, needs, and values, and ensuring that patient values guide all clinical decisions” and has been proven to be an important predictor for patient satisfaction and treatment outcomes [6].

One approach to assess these needs is to evaluate the patient’s health-related quality of life (HRQoL) during therapy. They can be measured with the help of questionnaires. A distinction is made between disease-specific and generic (cross-disease) quality of life (QoL) questionnaires [7]. The gold standard for recording HRQoL is patient-reported outcomes (PROs) [8]. PROs describe the collection of health and health care data by the patients themselves, without interpretation by physicians or other health care providers [9]. This information can include symptoms, functional status, QoL, and quality of care. As patients directly transmit these data, side effects and losses in QoL that are not queried or underestimated by the medical staff are recorded in an unfiltered process.

PROs have proven to be a useful tool both for detecting adverse events (AEs) in the context of studies and for investigating HRQoL, treatment, and care. In particular, in drug trials, PRO-AEs represent the gold standard for measuring side effects [10]. Studies have shown that practitioners generally underestimate a large proportion of the relevant side effects or do not notice them at all [11,12]. By recording PROs, precision in monitoring therapy can thus be increased. This more relevant information on treatment, in turn, contributes to improving therapy quality and patient satisfaction [13].

PROs can be collected electronically, generating information about health status in real time both for the patients themselves and for the treatment team and offering the possibility to communicate in both directions, that is, information for the treatment team and feedback to the patients [12,14]. This provides immediate feedback, and interventions can be established if the results of the ePRO survey prove to be abnormal or critical. Studies have shown that this improves symptom control [15,16], offers improved doctor-patient communication [17,18], and increases patient well-being and satisfaction [19,20]. Basch et al [12,21] attracted particular attention with their work on ePROs in patients with cancer, as he was able to show that electronic patient-reported outcome measurement not only significantly improves HRQoL but also reduces emergency room visits and even improves overall survival in patients with metastatic cancer disease [12,21]. These results were confirmed in another study in patients with advanced lung cancer [22].

In the context of patient-centered care, PROs can represent an important component in clinical routine, comparable to laboratory values, in order to give the patients’ HRQoL more weight in making decisions about treatment [7]. They can help stratify the side effects of the individual patient, make them more objective, and relate them to comparison groups.

Despite the positive outcomes of PRO-based interventions in patients undergoing metastatic (breast) cancer therapies, their impact in the neoadjuvant and adjuvant settings remains unclear. However, initial evidence suggests that PRO-supported monitoring may also have beneficial effects on HRQoL and in preventing AEs in these treatment contexts [23]. The available data, particularly regarding adjuvant therapy in patients with breast cancer, are still limited, and PRO-based interventions have not yet demonstrated a clinically significant effect [24].

The ENABLE study was designed to systematically evaluate HRQoL and AEs in patients with invasive breast cancer using digital questionnaires through the specially developed smartphone app “ENABLE.” By implementing targeted interventions, the study aimed to improve treatment satisfaction and HRQoL across various therapy settings. Additionally, it sought to examine the impact of PRO-based monitoring on patients receiving neoadjuvant and adjuvant chemotherapy and to determine whether findings from metastatic settings could be applied to these treatment phases.

## Methods

### Study Design

**Figure 1:**
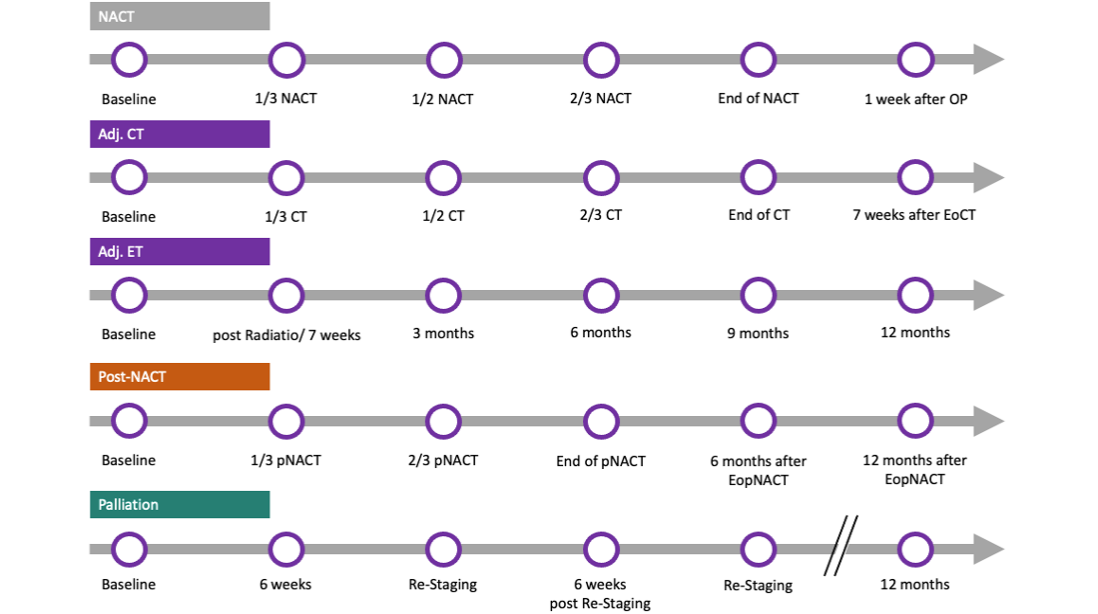
Timepoints of questionnaire assessments (visits) depending on the therapy and therapeutic strategy. Adj. CT: adjuvant chemotherapy; Adj. ET: adjuvant endocrine therapy; CT: chemotherapy; EoCT: end of chemotherapy; NACT: neoadjuvant chemotherapy; OP: operation; POST-NACT/pNACT: postneoadjuvant chemotherapy.

Patients were included before starting systemic therapy for breast cancer in neoadjuvant, adjuvant, postneoadjuvant, and palliative treatment settings. Patients were recruited at the respective oncology centers. The study randomized patients 1:1 into an intervention and a control group. In both groups, QoL was assessed for at least six fixed time points during therapy using European Organization for the Research and Treatment of Cancer Quality of Life Questionnaire-C30, European Organization for the Research and Treatment of Cancer Quality of Life Questionnaire-BR45, and Patient-Reported Outcomes Measurement Information System-29 (PROMIS-29; Figure 1). Participating centers are the university hospitals of Heidelberg, Tuebingen, and Mannheim, Germany.

### Intervention Group

**Figure 2:**
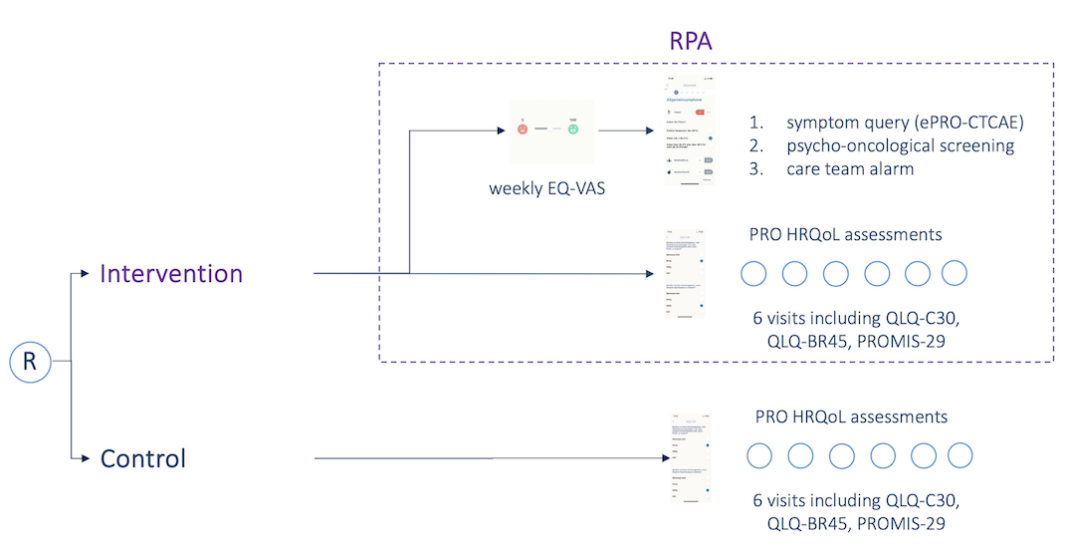
Study design of the ENABLE trial: upon patient enrollment in the care portal, randomization is performed automatically using centralized block randomization. ePRO-CTCAE: electronic patient-reported outcome Common Terminology Criteria of Adverse Event; HRQoL: health-related quality of life; PRO: patient-reported outcome; PROMIS-29: Patient-Reported Outcomes Measurement Information System-29; QLQ-BR45: European Organization for the Research and Treatment of Cancer Quality of Life Questionnaire-BR45; QLQ-C30: European Organization for the Research and Treatment of Cancer Quality of Life Questionnaire-C30; R: 1:1 randomization; RPA: reactive patient-reported outcome assessment.

**Figure 3:**
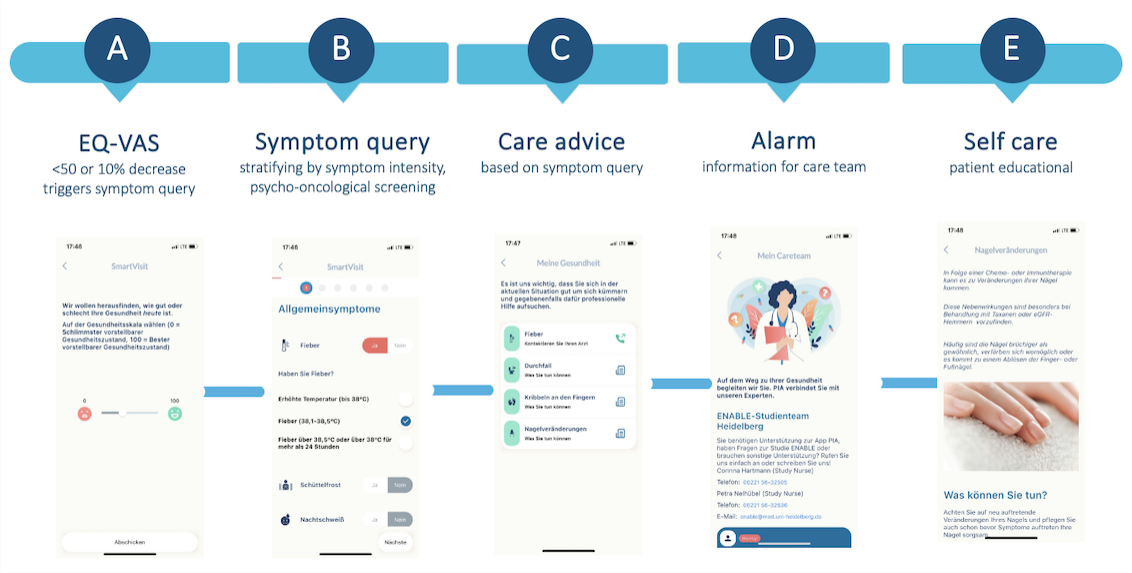
(A) RPA procedure with HRQoL screening via EQ-VAS, (B) symptom query, and psycho-oncological screening in case of a value <50 or a decrease of 10 % in comparison to the last EQ-VAS value. The symptom query is followed by (C) care advice, (D) an alarm for the care team via the “PIA” platform, and (E) educational content for the patient via the app. The images show the app representations for the patient. centralized block randomization. PRO-CTCAE: patient-reported outcome Common Terminology Criteria of Adverse Event; HRQoL: health-related quality of life; RPA: reactive patient-reported outcome assessment.

In the intervention group, a brief weekly assessment of the current HRQoL was additionally carried out via the app using a visual analog scale (EQ-VAS). The EQ-VAS was used as a screening tool, as previous research has demonstrated its sensitivity in detecting deterioration of HRQoL at an early stage [25]. If significant deterioration (EQ-VAS value <50 or a decrease of 10% in comparison to the last EQ-VAS value) in the current HRQoL was discovered in the intervention group, a digital questionnaire was started, which asked about therapy-associated side effects in a graduated manner (ePRO symptom query based on the Common Terminology Criteria of Adverse Events stratification), that is, classified according to symptom severity, and the treatment team was informed; this so-called reactive PRO assessment (RPA) is depicted in Figure 2. Furthermore, a graduated psychological assessment was conducted using Patient Health Questionnaire-8, Generalized Anxiety Disorder 7, and the European Organization for Research and Treatment of Cancer Quality of Life Questionnaire for Cancer-related Fatigue. In addition, the patients were presented with evidence-based educational information via the app on how to deal with the side effects as learning content, and if the side effects were more severe, they were asked to contact the treatment team. In parallel, the treatment team was informed via a web app (“PIA”), and depending on the severity of the complaints, the patient was contacted directly. Figure 3 illustrates the RPA procedure.

### Patient Empowerment

Each patient had an individual treatment and care plan that was mapped via the app. Via a content library in the app, all study participants were provided with information about care offers, such as self-care practices for managing side effects, nutritional counseling, social counseling, physiotherapy, and sport offers, and with patient-centered, evidence-based educational information. Depending on the respective therapy and the individual situation of the patients, this information was displayed as a feed on the app’s home screen. In addition, the app provided an overview of the current status of the therapy and displayed the next appointments at the center via a calendar.

**Figure 4:**
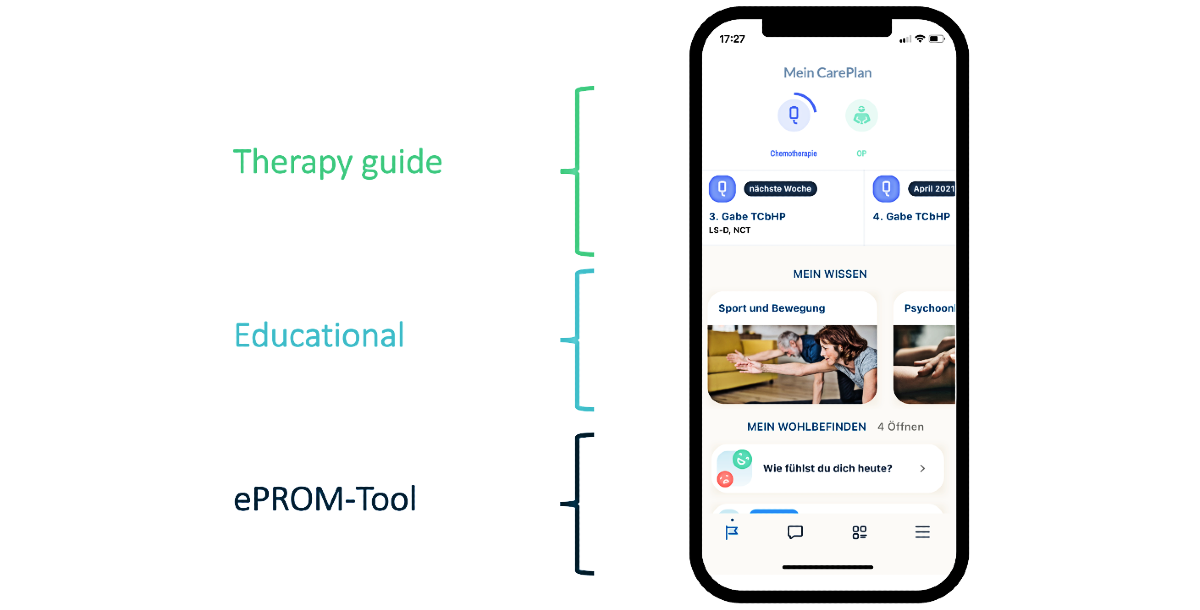
The home screen of the ENABLE app with representation of the different functions. ePROM: electronic patient-reported outcome measurement.

The smartphone app “ENABLE” was thus a bidirectional communication platform with an additional educational guide. This enabled patient education, and thus, patient empowerment on the one hand, and risk assessment, therapy control, and systematic QoL assessment on the other (Figure 4). The questionnaires and guides were individually adapted to the respective therapy situation and patient needs.

### Care Team Web App

The treatment team of the respective study center had access to the web-based care team platform “PIA,” developed and provided by the Center for Innovative Care and the Institute of Women’s Health GmbH, via which the patients were included in the study and assigned to a treatment and care plan. The PRO data for each patient were visualized via the platform and alarms in the intervention group were displayed. In addition, the calendar could be set and the clinical data entered.

### Aim

The study aims to improve therapy adherence, recognize and treat critical side effects in a timely manner, and make the HRQoL more objective in the context of different therapy strategies. The study is intended to test the overall benefit and added value of an electronic therapy companion and to increase patient satisfaction.

### Primary Hypothesis and Outcome

The primary end point of the study is to compare HRQoL and treatment satisfaction (measured by the PROMIS-29) between the intervention and control groups.

### Secondary Hypotheses and Outcomes

Participation in the intervention group is expected to improve treatment satisfaction, therapy adherence, and detection and timely treatment of critical AEs. The close-meshed, weekly, brief HRQoL assessment was tested as a screening tool to detect relevant side effects during therapy. The study provides HRQoL objectivity across treatment strategies. In addition, a usability and feasibility analysis of the app and the intervention concept was carried out.

### Inclusion Criteria

The inclusion criteria are as follows: diagnosis of invasive breast cancer or metastasis of breast carcinoma; 18 years or older; fluency in German; access to a computer, tablet, or smartphone with internet connectivity; capacity to provide informed consent; and a signed informed consent form.

### Data Acquisition and Management

The study is based on the principles of the Declaration of Helsinki. The study plan and all other study-relevant documents were approved by the ethics committees of the university medical faculties of Heidelberg, Mannheim, and Tuebingen, Germany. The current version of the study protocol is 4.2 from December 9, 2022. Protocol amendments were communicated to participating centers immediately. The study participants have the right to request information about the personal data collected from them. Participation in the study is voluntary after all study content and objectives have been declared and written consent has been given and can be terminated at any time without any disadvantages for the patient.

Patient data and clinical data are recorded pseudonymously and stored centrally in the electronic case report form (eCRF). The data were documented from the medical reports and tumor documentation of the treating center in a web-based eCRF via the study staff at the study centers. These eCRFs are stored and managed throughout the duration of the study in a secured cloud infrastructure, which was conceptualized and initiated by the Institute of Women’s Health GmbH and the three partner sites of the Center for Innovative Care in Heidelberg, Mannheim, and Tuebingen. This high-availability infrastructure guarantees and verifies individual permission rights and user roles for all participating clinicians for strict compliance with data protection regulations. For future research, all accumulated data will be transferred into the individual clinic infrastructures after the study ends, and all externally hosted data will be permanently erased. An interim analysis of the secondary end points was conducted after 50% of the patients had been included and carried out by the Institute of Women’s Health GmbH. The data monitoring committee is made up of the principal investigators from the Heidelberg and Tuebingen centers and is independent of the sponsor of the study.

The following clinical data are recorded: demographic data, Eastern Cooperative Oncology Group status, height, weight, stage of the disease, previous diseases, clinical tumor characteristics, tumor biology, staging results, therapy-associated side effects, and previous and current therapies.

### Sample Size Calculation

The sample size was calculated according to the primary end point HRQoL (measured via the PROMIS-29). It was based on a minimal important difference of 5 points, an SD of 10 points, a power of 80%, a 2-sided significance level of 5%, and a dropout rate of 37.5%. PASS (NCSS, LLC) software was used to calculate the sample size.

### Statistical Analysis

The primary outcomes will be examined in an intention-to-treat analysis for all randomized individuals with a mixed linear regression model. Primary analysis will be adjusted for age, therapy situation, type of systemic therapy, Eastern Cooperative Oncology Group status at enrollment, and other important confounders if necessary. The heterogeneity between the trial sites is considered through random effects (random intercept mixed model). The level of significance is set at α=5%.

All secondary outcomes will be analyzed analogously to the primary hypothesis and according to the respective outcome using further suitable statistical methods. As in the analysis of the primary outcome, the secondary hypotheses will be adjusted for relevant confounders. In addition, the heterogeneity between study centers will be considered. Secondary outcome analysis will be performed exploratively.

All statistical analyses will be planned in detail and documented in a statistical analysis plan that will be prepared before closing the database.

### Ethical Considerations

The study was approved by the central ethics committee in Heidelberg (S-658/2020). All recruiting centers obtained approval from the local ethics committees (Ethics Committee at the Medical Faculty of the Eberhard Karls University of Tuebingen and the University Hospital of Tuebingen: 896/2020BO2; Ethics Committee II of the University of Heidelberg, Mannheim Medical Faculty: 2021-544) before study initiation. Written informed consent was obtained from all participants before study participation. The authors affirm that human research participants provided informed consent for publication. Patients signed informed consent regarding publishing their data. Participants received no financial or other compensation for their involvement in the study.

## Results

Recruitment started on March 22, 2021, in Heidelberg; April 11, 2021, in Tuebingen; and November 4, 2021, in Mannheim. The last patient was enrolled on September 8, 2023, and the last visit was in February 2024. Due to the innovative specifications of the app, the protocol was only published after the recruitment phase was completed. The protocol was submitted before the end of data collection. A total of 606 patients were enrolled in the study; however, 14 patients did not complete the app’s initial setup process and were therefore excluded from the trial. The remaining 592 patients included in the trial consisted of 380 (64.2%) patients who received neoadjuvant chemotherapy, 138 (23.3%) patients who underwent adjuvant chemotherapy, 38 (6.4%) patients who received adjuvant endocrine therapy, 12 (2%) patients who underwent postneoadjuvant therapy, and 4 (0.7%) patients who received palliative chemotherapy. The first results are expected to be published in Q2 2025.

## Discussion

### Principal Findings

Study results published in the last few years have already demonstrated the many possibilities for increasing the quality of care, treatment, and HRQoL in breast cancer treatment due to patient-reported outcome measurements (PROMs), and survival rates have improved, too [21,22]. These results fundamentally underscore the necessity of consistently integrating eHealth and telemedicine, particularly in the context of electronic patient-reported outcome measurement, into breast cancer therapy.

However, some barriers to implementing eHealth in mainstream care remain. Among the theoretical aspects is the fact that no standard has been established yet either for selecting questionnaires or for downstream interventions. One task for the coming years will be to develop basic items and a basic dataset in order to establish a system for quality control and to make it possible to compare the collected data.

For the purpose of standardizing the PROMs for a value-based PRO, the “International Consortium for Health Outcomes Measurement” has defined a minimal standard PRO Set [26]. In addition, the “Innovative Medicines Initiative—Health Outcomes Observatory” (H2O) consortium has developed core outcome sets to make HRQoL in patients with metastatic breast cancer more objective [27]. However, there is still no standard PROMs for PRO-based interventions in oncology.

In principle, we need to determine which intervals between assessments and for how long eHealth monitoring—in the sense of a PROM—are useful for patients with breast cancer. These questions need to be addressed in clinical studies, while simultaneously striving to integrate the findings from individual studies. Only then can PROMs be sensibly implemented in standard care. At the same time, different apps and providers must be able to access these interfaces, and we must also be able to transfer downstream interventions to a common system. Otherwise, implementation in everyday life could fail due to the abundance of additional documentation work required and the cost of developing additional software and apps.

The ongoing digitalization of all areas of life will ensure that there is no way back to less digitalized health care. However, despite all considerations of progress, the patient must never be left out of the focus of these efforts. Digitalization and the use of eHealth must always be based first and foremost on improving patient care and should never be an end in itself. The possibility to aggregate data through eHealth apps must also be weighed very critically in terms of scientific benefit and burden for the patient.

The ENABLE study aims to investigate the impact of a PRO-based intervention in different treatment settings for breast cancer. Recently, Karsten [28] presented findings at the San Antonio Breast Cancer Symposium demonstrating the beneficial effects of PRO-based interventions on fatigue, physical functioning HRQoL, and overall survival in the metastatic setting, too, underscoring the importance of bringing PRO monitoring into the standard of care for advanced breast cancer. These findings align with other positive PRO intervention studies in metastatic breast cancer that found improved therapy adherence, delayed onset of serious AEs, reduced toxicity, fewer unplanned hospitalizations, increased patient satisfaction, and enhanced HRQoL [29-31].

### Conclusions

The impact of neoadjuvant and adjuvant chemotherapy on HRQoL has not yet been thoroughly investigated. It is particularly important to explore the extent to which a PRO-based intervention may also positively influence patients’ HRQoL and symptoms in the adjuvant setting. Pappot et al [24] could not show a significant impact of a PRO-based intervention on treatment adjustments, unplanned hospitalization, or febrile neutropenia in a cohort of patients with breast cancer undergoing adjuvant therapy. Therefore, beyond its primary end point, the ENABLE study offers the opportunity to examine the effects of the intervention and the HRQoL on therapy outcomes, adherence, and AEs.

## Abbreviations

AE: adverse event
eCRF: electronic case report form
HRQoL: health-related quality of life
PRO: patient-reported outcome
PROM: patient-reported outcome measurement
PROMIS-29: Patient-Reported Outcomes Measurement Information System-29
QoL: quality of life
RPA: reactive patient-reported outcome assessment

## Data Availability

Data sharing is not applicable to this article as no datasets were generated or analyzed.

## References

[1] Bray F, Ferlay J, Soerjomataram I, Siegel RL, Torre LA, Jemal A (2018). Global cancer statistics 2018: GLOBOCAN estimates of incidence and mortality worldwide for 36 cancers in 185 countries. CA Cancer J Clin.

[2] Burstein H, Curigliano G, Thürlimann B, Weber WP, Poortmans P, Regan MM, Senn HJ, Winer EP, Gnant M (2021). Customizing local and systemic therapies for women with early breast cancer: the St. Gallen international consensus guidelines for treatment of early breast cancer 2021. Ann Oncol.

[3] de Melo Gagliato D, Lei X, Giordano S, Valero V, Barcenas C, Hortobagyi G, Chavez-MacGregor M (2020). Impact of delayed neoadjuvant systemic chemotherapy on overall survival among patients with breast cancer. Oncologist.

[4] Chavez-MacGregor M, Clarke CA, Lichtensztajn DY, Giordano SH (2016). Delayed initiation of adjuvant chemotherapy among patients with breast cancer. JAMA Oncol.

[5] Housten AJ, Malinowski C, Paredes E, Harris CL, McNeill LH, Chavez-MacGregor M (2022). Movement through chemotherapy delay to initiation among breast cancer patients: a qualitative analysis. Patient Prefer Adherence.

[6] Plewnia A, Bengel J, Körner M (2016). Patient-centeredness and its impact on patient satisfaction and treatment outcomes in medical rehabilitation. Patient Educ Couns.

[7] Schmidt M, Steindorf K (2021). [Quality of life after breast cancer: assessment, relevance and effective interventions]. TumorDiagn Ther.

[8] Basch E (2009). Patient-reported outcomes in drug safety evaluation. Ann Oncol.

[9] U.S. Department of HealthHuman Services FDA Center for Drug EvaluationResearch, U.S. Department of HealthHuman Services FDA Center for Biologics EvaluationResearch, U.S. Department of HealthHuman Services FDA Center for DevicesRadiological Health (2006). Guidance for industry: patient-reported outcome measures: use in medical product development to support labeling claims: draft guidance. Health Qual Life Outcomes.

[10] Basch E (2010). The missing voice of patients in drug-safety reporting. N Engl J Med.

[11] Basch EM, Reeve BB, Mitchell SA, Clauser SB, Minasian L, Sit L, Chilukuri R, Baumgartner P, Rogak L, Blauel E, Abernethy AP, Bruner D (2011). Electronic toxicity monitoring and patient-reported outcomes. Cancer J.

[12] Basch E, Deal AM, Kris MG, Scher HI, Hudis CA, Sabbatini P, Rogak L, Bennett AV, Dueck AC, Atkinson TM, Chou JF, Dulko D, Sit L, Barz A, Novotny P, Fruscione M, Sloan JA, Schrag D (2016). Symptom monitoring with patient-reported outcomes during routine cancer treatment: a randomized controlled trial. J Clin Oncol.

[13] Basch E (2014). New frontiers in patient-reported outcomes: adverse event reporting, comparative effectiveness, and quality assessment. Annu Rev Med.

[14] Basch E, Artz D, Dulko D, Scher K, Sabbatini P, Hensley M, Mitra N, Speakman J, McCabe M, Schrag D (2005). Patient online self-reporting of toxicity symptoms during chemotherapy. J Clin Oncol.

[15] Abernethy AP, Herndon JE, Wheeler JL, Day JM, Hood L, Patwardhan M, Shaw H, Lyerly HK (2009). Feasibility and acceptability to patients of a longitudinal system for evaluating cancer-related symptoms and quality of life: pilot study of an e/Tablet data-collection system in academic oncology. J Pain Symptom Manage.

[16] Kroenke K, Krebs EE, Wu J, Yu Z, Chumbler NR, Bair MJ (2014). Telecare collaborative management of chronic pain in primary care: a randomized clinical trial. JAMA.

[17] Berry DL, Blumenstein BA, Halpenny B, Wolpin S, Fann JR, Austin-Seymour M, Bush N, Karras BT, Lober WB, McCorkle R (2011). Enhancing patient-provider communication with the electronic self-report assessment for cancer: a randomized trial. J Clin Oncol.

[18] McCann L, Maguire R, Miller M, Kearney N (2009). Patients' perceptions and experiences of using a mobile phone-based advanced symptom management system (ASyMS) to monitor and manage chemotherapy related toxicity. Eur J Cancer Care (Engl).

[19] Schmidt ME, Wiskemann J, Steindorf K (2018). Quality of life, problems, and needs of disease-free breast cancer survivors 5 years after diagnosis. Qual Life Res.

[20] Chen J, Ou L, Hollis SJ (2013). A systematic review of the impact of routine collection of patient reported outcome measures on patients, providers and health organisations in an oncologic setting. BMC Health Serv Res.

[21] Basch E, Deal AM, Dueck AC, Scher HI, Kris MG, Hudis C, Schrag D (2017). Overall survival results of a trial assessing patient-reported outcomes for symptom monitoring during routine cancer treatment. JAMA.

[22] Denis F, Basch E, Septans A, Bennouna J, Urban T, Dueck AC, Letellier C (2019). Two-year survival comparing web-based symptom monitoring vs routine surveillance following treatment for lung cancer. JAMA.

[23] Maguire R, McCann L, Kotronoulas G, Kearney N, Ream E, Armes J, Patiraki E, Furlong E, Fox P, Gaiger A, McCrone P, Berg G, Miaskowski C, Cardone A, Orr D, Flowerday A, Katsaragakis S, Darley A, Lubowitzki S, Harris J, Skene S, Miller M, Moore M, Lewis L, DeSouza N, Donnan PT (2021). Real time remote symptom monitoring during chemotherapy for cancer: European multicentre randomised controlled trial (eSMART). BMJ.

[24] Pappot H, Baeksted CW, Nissen A, Knoop A, Mitchell SA, Christensen J, Hjollund NH, Johansen C (2021). Clinical effects of assessing electronic patient-reported outcomes monitoring symptomatic toxicities during breast cancer therapy: a nationwide and population-based study. Breast Cancer.

[25] Brusniak K, Feisst M, Sebesteny L, Hartkopf A, Graf J, Engler T, Schneeweiss A, Wallwiener M, Deutsch TM (2021). Measuring the time to deterioration for health-related quality of life in patients with metastatic breast cancer using a web-based monitoring application: longitudinal cohort study. JMIR Cancer.

[26] Ong WL, Schouwenburg MG, van Bommel AC, Stowell C, Allison KH, Benn KE, Browne JP, Cooter RD, Delaney GP, Duhoux FP, Ganz PA, Hancock P, Jagsi R, Knaul FM, Knip AM, Koppert LB, Kuerer HM, McLaughin S, Mureau MAM, Partridge AH, Reid DP, Sheeran L, Smith TJ, Stoutjesdijk MJ, Peeters MJTV, Wengström Y, Yip C, Saunders C (2017). A standard set of value-based patient-centered outcomes for breast cancer: the international consortium for health outcomes measurement (ICHOM) initiative. JAMA Oncol.

[27] de Ligt KM, de Rooij BH, Hedayati E, Karsten MM, Smaardijk VR, Velting M, Saunders C, Travado L, Cardoso F, Lopez E, Carney N, Wengström Y, Ives A, Velikova G, Fialho MDLS, Seidler Y, Stamm TA, Koppert LB, van de Poll-Franse LV (2023). International development of a patient-centered core outcome set for assessing health-related quality of life in metastatic breast cancer patients. Breast Cancer Res Treat.

[28] Karsten MM (2024). GS1-06: PRO B—a superiority randomized controlled trial evaluating the effects of symptom monitoring in metastatic breast cancer patients. San Antonio Breast Cancer Symposiom.

[29] Harbeck N, Kates R, Schinköthe T, Schumacher J, Wuerstlein R, Degenhardt T, Lüftner D, Räth P, Hoffmann O, Lorenz R, Decker T, Reinisch M, Göhler T, Staib P, Gluz O, Fasching PA, Schmidt M (2023). Favorable impact of therapy management by an interactive eHealth system on severe adverse events in patients with hormone receptor-positive, HER2-negative locally advanced or metastatic breast cancer treated by palbociclib and endocrine therapy. Cancer Treat Rev.

[30] Harbeck N, Fasching PA, Wuerstlein R, Degenhardt T, Lüftner D, Kates RE, Schumacher J, Raeth P, Hoffmann O, Lorenz R, Decker T, Reinisch M, Göhler T, Staib P, Gluz O, Schinkoethe T, Schmidt M (2023). CANKADO PRO-React eHealth support in patients with HR+ HER2- metastatic breast cancer receiving palbociclib and endocrine therapy and the affect on time to deterioration of quality of life: Primary outcome analysis of the multicenter randomized PreCycle trial. JCO.

[31] Mir O, Ferrua M, Fourcade A, Mathivon D, Duflot-Boukobza A, Dumont S, Baudin E, Delaloge S, Malka D, Albiges L, Pautier P, Robert C, Planchard D, de Botton S, Scotté F, Lemare F, Abbas M, Guillet M, Puglisi V, Di Palma M, Minvielle E (2022). Digital remote monitoring plus usual care versus usual care in patients treated with oral anticancer agents: the randomized phase 3 CAPRI trial. Nat Med.

