# Correction: ENABLE—App-Based Digital Capture and Intervention of Patient-Reported Quality of Life, Adverse Events, and Treatment Satisfaction in Breast Cancer: Protocol for a Randomized Controlled Trial

**DOI:** 10.2196/78781

**Published:** 2025-06-11

**Authors:** Thomas M Deutsch, Léa L Volmer, Manuel Feisst, Laura Bodenbeck, Kathrin Hassdenteufel, Lara Tretschock, Christiane Breit, Stefan Stefanovic, Armin Bauer, Carolin Anders, Lina Weinert, Tobias Engler, Andreas D Hartkopf, Nico Pfeifer, Pascal Escher, Marc Mausch, Oliver Heinze, Marc Suetterlin, Sara Y Brucker, Andreas Schneeweiss, Markus Wallwiener

**Affiliations:** 1 Department of Gynecology and Obstetrics Heidelberg University Hospital Heidelberg Germany; 2 Department of Gynecology and Obstetrics University Hospital Tuebingen Tuebingen Germany; 3 Institute of Medical Biometry Heidelberg University Heidelberg Germany; 4 Department of Gynecology and Obstetrics University Hospital Mannheim, Heidelberg University Mannheim Germany; 5 Institute Women's Health Tuebingen Germany; 6 Institute of Medical Informatics Heidelberg University Heidelberg Germany; 7 Directorate of Nursing Heidelberg University Hospital Heidelberg Germany; 8 Heidelberg Institute of Global Health, Section for Oral Health Heidelberg University Heidelberg Germany; 9 Department of Computer Science University of Tuebingen Tuebingen Germany; 10 Center for Innovative Care University Hospital Tuebingen Tuebingen Germany; 11 Institute for Bioinformatics and Medical Informatics University of Tuebingen Tuebingen Germany; 12 Rhein Main University of Applied Sciences Wiesbaden Germany; 13 National Center for Tumor Diseases University Hospital Heidelberg Heidelberg Germany; 14 German Cancer Research Center Heidelberg Germany; 15 Department of Gynecology and Obstetrics University Hospital Halle Halle Germany

In “ENABLE—App-Based Digital Capture and Intervention of Patient-Reported Quality of Life, Adverse Events, and Treatment Satisfaction in Breast Cancer: Protocol for a Randomized Controlled Trial” (JMIR Res Protoc 2025;14:e69855) the authors noted one error.

The following affiliation of authors Carolin Anders and Lina Weinert:

"Rhein Main University of Applied Sciences, Wiesbaden, Germany"

Has been revised to:

"Institute of Medical Informatics, Heidelberg University, Heidelberg, Germany"

The correction will appear in the online version of the paper on the JMIR Publications website together with the publication of this correction notice. Because this was made after submission to PubMed, PubMed Central, and other full-text repositories, the corrected article has also been resubmitted to those repositories.

